# Laparoscopic Management of a Complex Adrenal Cyst

**DOI:** 10.1155/2015/234592

**Published:** 2015-11-08

**Authors:** Koichi Kodama, Yasukazu Takase, Susumu Niikura, Akiko Shimizu, Hiroki Tatsu, Katsuhiko Saito

**Affiliations:** ^1^Department of Urology, Toyama City Hospital, Toyama, Toyama 939-8511, Japan; ^2^Department of Urology, Toyama Rosai Hospital, Uozu, Toyama 937-0042, Japan; ^3^Department of Internal Medicine, Toyama City Hospital, Toyama, Toyama 939-8511, Japan; ^4^Department of Radiology, Toyama City Hospital, Toyama, Toyama 939-8511, Japan; ^5^Department of Pathology, Toyama City Hospital, Toyama, Toyama 939-8511, Japan

## Abstract

Adrenal cysts are rare, and their clinical management remains controversial. We report a case involving an adrenal cyst with a complicated appearance on radiological studies. Unenhanced computed tomography revealed a unilocular, noncalcified, hypoattenuating mass with a thin wall in the left adrenal gland. The lesion gradually increased in size from 10 to 50 mm at two-year follow-up. On contrast-enhanced magnetic resonance imaging, a mural nodule with contrast enhancement was observed. The entire adrenal gland was excised *en bloc* via a lateral transperitoneal laparoscopic approach without violating the principles of surgical oncology. The pathological diagnosis was an adrenal pseudocyst. Laparoscopic adrenalectomy is a safe option for the treatment of complex adrenal cysts, while maintaining the benefits of minimal invasiveness.

## 1. Introduction

Cystic adrenal lesions represent a rare entity, and their clinical management remains controversial. It is possible to observe asymptomatic, nonfunctioning, benign-appearing adrenal cysts. However, the rubric of complex adrenal cystic lesions comprises a broad differential diagnosis, which renders definitive diagnosis and subsequent management difficult. This report describes a case involving an adrenal pseudocyst that was successfully treated using laparoscopic adrenalectomy without violating the principles of surgical oncology.

## 2. Case Presentation

**Figure 1:**
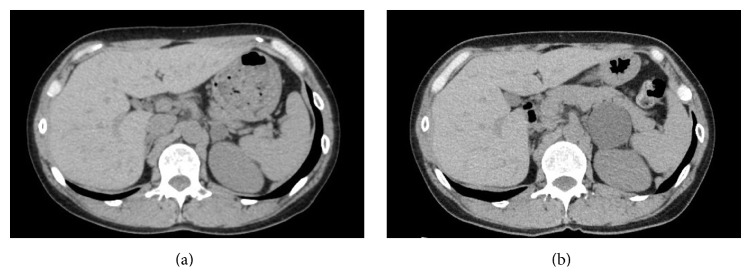
Unenhanced computed tomography of the abdomen showing a unilocular, noncalcified, hypoattenuating left adrenal lesion. The lesion increased in size from (a) 10 mm to (b) 50 mm at two-year follow-up.

**Figure 2:**
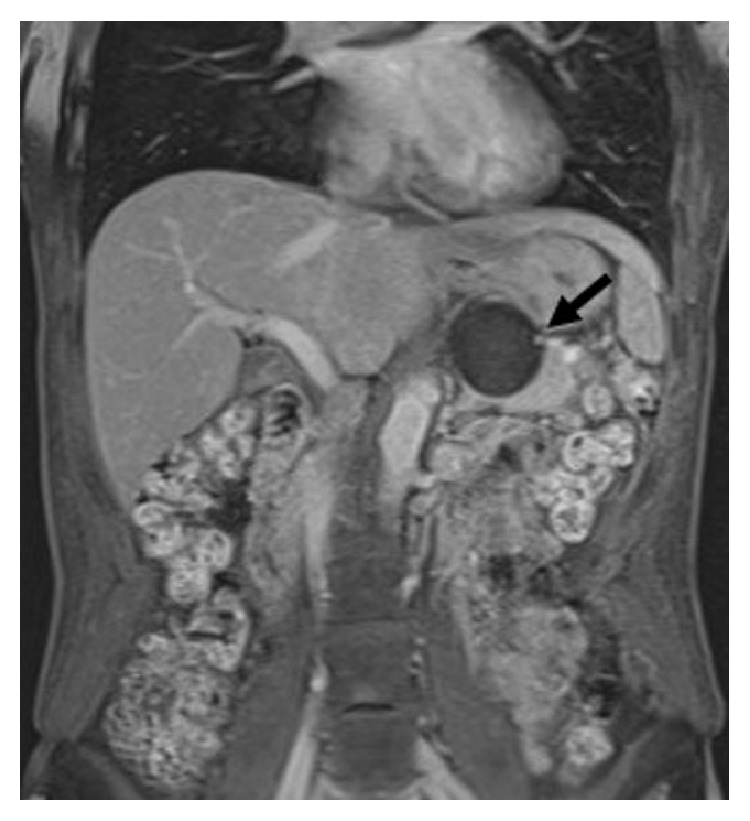
Axial T1-weighted magnetic resonance imaging showing homogeneous low signal intensity of the adrenal cystic lesion. A mural nodule with contrast enhancement (arrow) is observed.

A healthy 42-year-old woman presented to our institution with a right renal mass. Radiological investigations revealed an 8 cm heterogeneous soft tissue mass in the lower pole of the right kidney. The patient then underwent a transperitoneal laparoscopic nephrectomy of the right kidney with preservation of the ipsilateral adrenal gland. The pathology report of the surgical specimen indicated clear cell carcinoma, Fuhrman grade 2, T3aN0M0. On follow-up seven years after the nephrectomy, the patient remained asymptomatic, but unenhanced computed tomography of the abdomen revealed a 10 × 10 mm, unilocular, noncalcified mass with a thin wall in the left adrenal gland (Figure 1(a)). The internal density was homogeneously low and similar to water in attenuation. After the initial detection of the left adrenal lesion, the lesion gradually increased in size from 10 to 50 mm at 2-year follow-up (Figure 1(b)). On contrast-enhanced magnetic resonance imaging, the cystic lesion exhibited homogeneous low signal intensity on T1-weighted images and high signal intensity on T2-weighted images, and a mural nodule was found with contrast enhancement (Figure 2). Plasma cortisol, renin, and aldosterone levels were normal. A 24 h urine analysis revealed normal levels of catecholamines, vanillylmandelic acid, and metanephrines.

**Figure 3:**
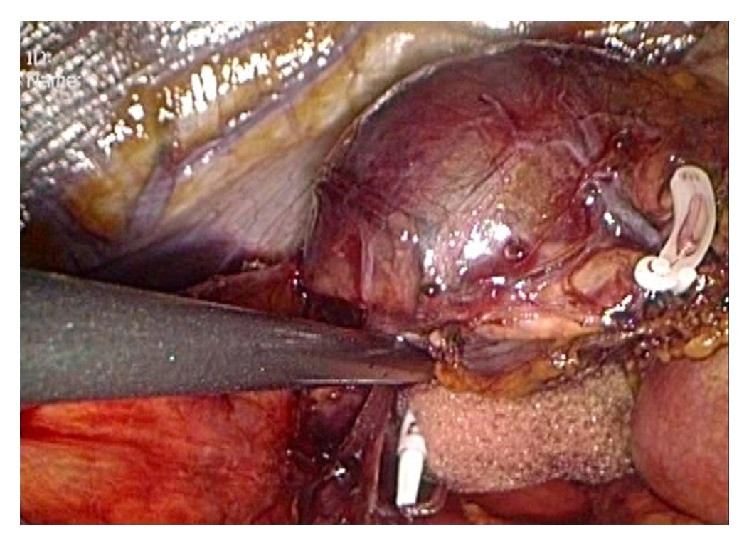
Intraoperative view. The entire adrenal gland including the cystic lesion is gently retracted upward with an endoscopic surgical spacer to avoid inadvertent cyst puncture.

**Figure 4:**
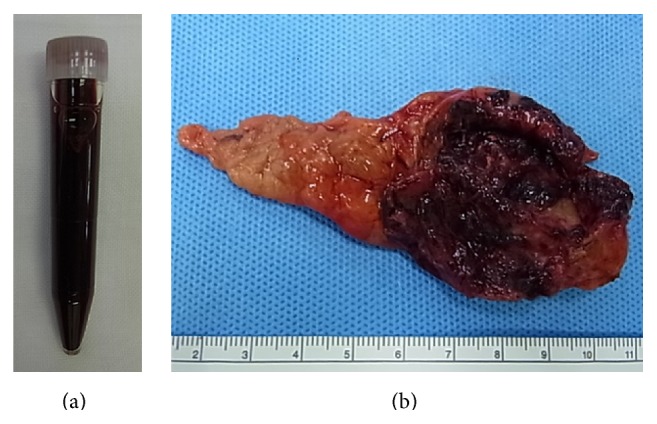
Photographs of (a) the blood-stained fluid from the cyst and (b) surgical specimen of the left adrenal gland including the remaining cyst components.

Laparoscopic adrenalectomy with four ports was performed using a lateral transabdominal approach. The cystic lesion had smooth boundaries and did not seem to be discriminated from the surrounding benign-appearing adrenal tissue. An endoscopic surgical spacer (Securea, Hogy Medical Co., Ltd., Tokyo, Japan), a polyurethane sponge with a radiopaque marker, was introduced into the abdominal cavity through a 12 mm trocar. The spacer was gripped with laparoscopic forceps to extend the adrenal gland including the cystic lesion, thereby ensuring a safe surgical field and avoiding inadvertent cyst disruption (Figure 3). The entire adrenal gland, including the cystic lesion, was excised* en bloc* and transferred into a laparoscopic retrieval bag (Endo Catch Gold 10 mm Specimen Pouch, Covidien, Mansfield, MA, USA). After protecting the exterior abdominal surface, the opening of the bag was pulled out of the camera port site, and an 18-gauge needle with a syringe was placed into the cyst to siphon all the fluid content (40 mL of blood-stained fluid) (Figure 4(a)). Subsequently, the remaining cyst components and the adrenal gland were removed with the bag, without enlargement of the original trocar incision. All surgical procedures performed strictly adhered to the principles of surgical oncology. Intraoperative blood loss was minimal, and the postoperative period was uneventful. The patient was discharged on day 3.

The gross appearance of the surgical specimen revealed red-tan friable tissue, consistent with internal hemorrhage (Figure 4(b)). On histopathological examination, the cyst wall contained no endothelial or epithelial lining. The pathological diagnosis was an adrenal pseudocyst. Neither recurrence nor adrenal insufficiency has been observed during six months of follow-up.

## 3. Discussion

With the advent and widespread use of imaging studies, adrenal cystic lesions are currently more commonly diagnosed. They are incidentally discovered on imaging examinations, with a prevalence of 1% reported in a large series of 1049 adrenal lesions [1]. Foster modified and histopathologically classified 220 adrenal cysts into the following four types: (a) parasitic cysts (7%), (b) epithelial cysts (9%), (c) pseudocysts (39%), and (d) endothelial cysts (45%) [2]. In several immunohistochemical studies, the latter two types of adrenal cysts were described as variants of vascular cysts. Although various theories have been proposed regarding the pathogenesis of adrenal vascular cysts, they probably originate from a preexisting vascular hamartoma [3].

Management algorithms for adrenal cysts vary and are controversial because of the overall rarity of such lesions. The rubric of complex adrenal cystic lesions comprises a broad differential diagnosis, which renders definitive diagnoses difficult. Adrenal cysts constitute clinical entities with a malignant potential rate of approximately 7% [4]. In a current report, changes in adrenal cyst size (either increase or decrease) are not unusual, and an increase in size does not indicate malignancy [5]. The internal texture, density, wall thickness, calcification pattern, and absence of true contrast enhancement should be thoroughly assessed when diagnosing adrenal cysts on the basis of radiological studies. Adrenal pseudocysts are typically unilocular cystic lesions, and they may appear as mixed or even solid masses because of fresh or organized hematomas [6]. Depending on the stage of evolving hematoma, adrenal pseudocysts may have a complicated appearance, manifesting with septations, blood products, fluid-fluid levels, or soft tissue components [7].

Surgical excision of adrenal cysts is indicated by the presence of symptoms, suspicion of malignancy, or the detection of a functioning adrenal cyst. Fine-needle-aspiration biopsy cytology of the cystic lesion has been recommended as a method of differentiating benign cysts of the adrenal gland from malignant cysts [8]. Laparoscopic unroofing of the cysts appears to be appropriate for managing symptomatic benign adrenal cysts that have smooth walls and homogeneous, clear contents [9]. However, those procedures have the potential risk of spreading neoplastic cells if the tumor is malignant. The indications for partial adrenalectomy have yet to be defined along with technical aspects such as the extent of excision and preservation of the adrenal vein. Therefore,* en bloc* adrenalectomy represents the appropriate procedure in cases involving complex adrenal cysts, such as the present case.

For radical excision of a complex adrenal cyst, the principles of surgical oncology cannot be overemphasized. Laparoscopic adrenalectomy has several benefits, such as smaller incisions, shorter hospital stays, and faster recovery; however, open adrenalectomy for adrenal cystic tumors for which there was suspicion of malignancy still accounts for most cases reported in the literature. Few articles have described laparoscopic adrenalectomy for complex adrenal cysts [10–12]. The reason open surgery was chosen most often was probably because of the expected difficulties in dissecting out and removing the cystic tumor* en bloc* from small wounds; we can overcome these difficulties using our technique. To dissect the cystic tumor, Securea was useful for achieving complete tumor resection without violating the cystic wall. Furthermore, to remove the large cystic tumor* en bloc* from small wounds, we used the surgical techniques described by Hung et al. [10], which ensured intact removal of the adrenal cystic tumor through a small port wound without contaminating the intra-abdominal structures or incision site. Further studies are necessary to evaluate the benefits and safety of laparoscopic surgery compared with those of open surgery to treat complex adrenal cysts.

Adrenal pseudocysts may rapidly increase in size and exhibit a complicated appearance in radiological images. Laparoscopic adrenalectomy is a safe option for the treatment of complex adrenal cysts, while maintaining the benefits of minimal invasiveness.
